# Differential proteome analysis of human embryonic kidney cell line (HEK-293) following mycophenolic acid treatment

**DOI:** 10.1186/1477-5956-9-57

**Published:** 2011-09-20

**Authors:** Muhammad Qasim, Hazir Rahman, Michael Oellerich, Abdul R Asif

**Affiliations:** 1 Department of Clinical Chemistry, University Medical Centre Goettingen, 37075, Goettingen, Germany; 2 Department of Microbiology, Kohat University of Science and Technology, 26000, Kohat, Pakistan

**Keywords:** HEK-293 cells, proteome, mycophenolic acid, drug toxicology, differential proteomics

## Abstract

**Background:**

Mycophenolic acid (MPA) is widely used as a post transplantation medicine to prevent acute organ rejection. In the present study we used proteomics approach to identify proteome alterations in human embryonic kidney cells (HEK-293) after treatment with therapeutic dose of MPA. Following 72 hours MPA treatment, total protein lysates were prepared, resolved by two dimensional gel electrophoresis and differentially expressed proteins were identified by QTOF-MS/MS analysis. Expressional regulations of selected proteins were further validated by real time PCR and Western blotting.

**Results:**

The proliferation assay demonstrated that therapeutic MPA concentration causes a dose dependent inhibition of HEK-293 cell proliferation. A significant apoptosis was observed after MPA treatment, as revealed by caspase 3 activity. Proteome analysis showed a total of 12 protein spots exhibiting differential expression after incubation with MPA, of which 7 proteins (complement component 1 Q subcomponent-binding protein, electron transfer flavoprotein subunit beta, cytochrome b-c1 complex subunit, peroxiredoxin 1, thioredoxin domain-containing protein 12, myosin regulatory light chain 2, and profilin 1) showed significant increase in their expression. The expression of 5 proteins (protein SET, stathmin, 40S ribosomal protein S12, histone H2B type 1 A, and histone H2B type 1-C/E/F/G/I) were down-regulated. MPA mainly altered the proteins associated with the cytoskeleton (26%), chromatin structure/dynamics (17%) and energy production/conversion (17%). Both real time PCR and Western blotting confirmed the regulation of myosin regulatory light chain 2 and peroxiredoxin 1 by MPA treatment. Furthermore, HT-29 cells treated with MPA and total kidney cell lysate from MMF treated rats showed similar increased expression of myosin regulatory light chain 2.

**Conclusion:**

The emerging use of MPA in diverse pathophysiological conditions demands in-depth studies to understand molecular basis of its therapeutic response. The present study identifies the myosin regulatory light chain 2 and peroxiredoxin 1 along with 10 other proteins showing significant regulation by MPA. Further characterization of these proteins may help to understand the diverse cellular effects of MPA in addition to its immunosuppressive activity.

## Introduction

Mycophenolic acid (MPA) is a frequently used immunosuppressant for the prevention of acute rejection in patients undergoing allogenic renal, cardiac, lung, and liver transplantations [1,2]. MPA is a selective, reversible and uncompetitive inhibitor of inosine monophosphate dehydrogenase (IMPDH), a key regulatory enzyme in the *de novo *pathway of purine synthesis. It exhibits cytotoxic effects on most of the cell types, but exerts greater effects on T and B lymphocytes, thus preventing solid organ rejection [2]. IMPDH inhibition by clinically relevant concentration of MPA results in guanine nucleotide depletion which is associated with G1 cell cycle arrest. MPA also triggers apoptosis by up-regulating pro-apoptotic proteins (p53, p21 and bax) and down-regulating proteins that are important for cell cycle progression, such as bcl-2, survivin p27 and c-myc [3]. IMPDH type II is significantly over-expressed in several tumor cells, for this reason IMPDH could be considered as a potent target for anti-cancer therapy, as well as immunosuppressive chemotherapy [4].

MPA and its metabolites effect most of the cellular functions by influencing biological pathways, like apoptosis [5], immune associated signaling [6] and general cell signaling pathways involving mitogen-activated protein kinases, extracellular-signal regulated kinases, c-Jun N-terminal kinases, p53 and Rho-associated protein kinase [5,7,8]. Collectively, MPA possesses anti-microbial, anti-inflammatory, anti-fibrotic, pro-apoptotic [2], anti-angiogenic, anti-cancerous [9] and anti-oxidant activities [10]. Due to MPA diverse therapeutic activities in the cell, it is also used for the treatment of dermatological diseases, neuromuscular diseases and autoimmune disorders such as lupus [9,11]. Gastrointestinal tract (GIT) complications i.e., diarrhoea, nausea, abdominal pain, vomiting, anorexia, gastritis, intestinal ulceration and small intestinal villous atrophy are common complication for some transplant patients on MPA therapy. Other MPA associated adverse effects are anemia, myelosuppression and risk of opportunistic infections [12]. The exact molecular mechanism of MPA organ toxicity is unknown, but possible mechanisms include direct toxicity by its anti-proliferative effect, opportunistic infections due to myelosuppression and toxicity, and acyl MPA glucuronide (AcMPAG) proteins adduct formation [12,13].

Here we use HEK-293 cell line to uncover cellular protein response to the exposure of clinical dose of MPA. In the present study we used a proteomics based approach to resolve proteins of total cell lysates on two dimensional electrophoresis (2-DE) gels following treatment with DMSO and MPA. The differentially expressed proteins were in-gel tryptic digested and identified by QTOF-MS/MS analysis. Several proteins were identified with modified expression in response to MPA treatment which might be helpful to broaden our understanding regarding the cellular effects of MPA.

## Results

**Figure 1:**
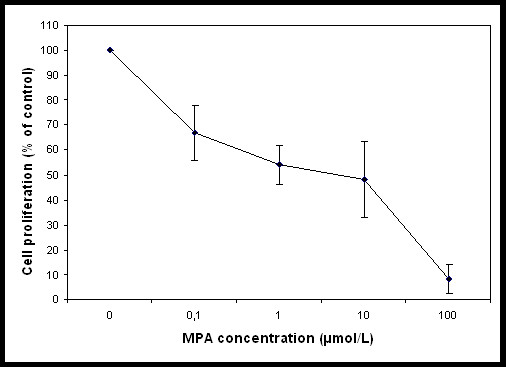
**Inhibition of HEK-293 cells proliferation by MPA treatment**. The cell proliferation was determined after 72 hr of treatment with different doses of MPA (0-100 μmol/L) using BrdU colorimetric based method. Results are shown as percentage of control (DMSO treated) and represent four independent experiments.

**Figure 2:**
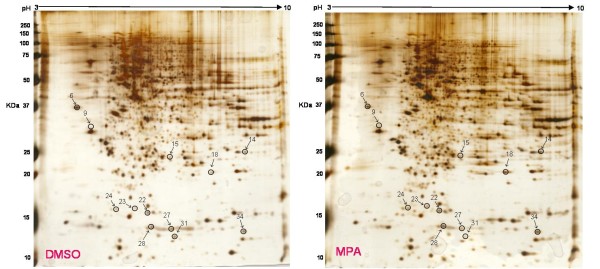
**Differential protein expression after incubation of HEK-293 cells with MPA**. Total protein lysate from DMSO and MPA treated cells was separated by 2-D gel electrophoresis and silver stained. Encircled differentially regulated proteins spots were identified using Q-TOF MS/MS analysis. The figure shows exemplary 2-DE gels of DMSO and MPA treated HEK-293 cells.

**Figure 3:**
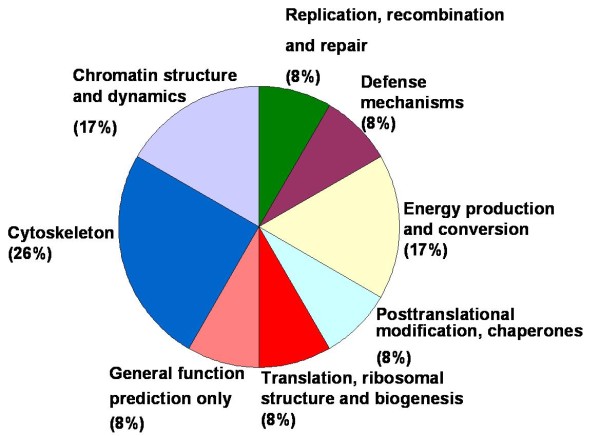
**Functional classification of regulated proteins**. Biological functions were assigned using online KOGnitor NCBI (http://www.ncbi.nlm.nih.gov/COG/grace/kognitor.html) software.

**Figure 4:**
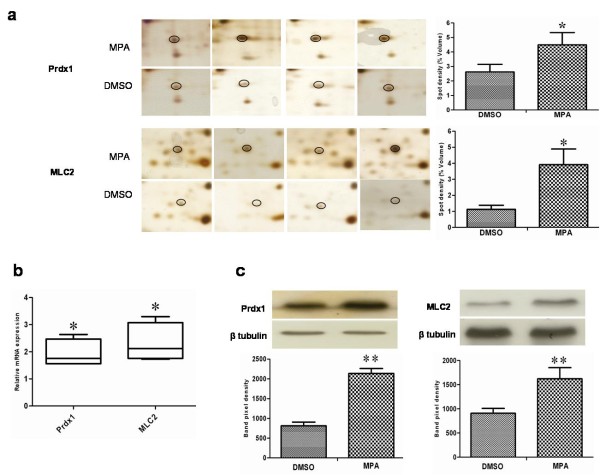
**Differential expression of Prdx1 and MLC2 by MPA treatment**. **(a) **Selected areas in the silver stained gels showing differential expression of Prdx1 and MLC2. Delta 2D software was used for densitrometric analysis. The quantification of the level of expression (% volume) in MPA treated cells and control cells (DMSO) is illustrated as a bar chart with the mean and SD of four separate experiments (*p < 0.05). **(b) **Expression patterns of Prdx1 and MLC2 genes determined by real-time PCR. The relative expression of Prdx1 and MLC2 mRNA in the treated samples was determined as a fold change compared with control samples using the comparative threshold cycle (C_T_) method (2^-ΔΔC^_T_) as described in materials and methods part. Results shown are representative of four independent experiments. EF-2 was used to normalize the values. The boxes represent range in variation statistics and the lines across the boxes represent the medians and the whiskers extend to the highest and lowest values. Significance was calculated using the Mann-Whitney-U test (*p < 0.05) **(c) **Effect of MPA treatment on Prdx1 and MLC2 protein expression. Protein extracts from MPA and DMSO treated cells were Western blotted using specific antibodies against Prdx1 and MLC2. Densitometric analysis was done using Lab image version 2.71 software. β tubulin signal was used to control the equal protein load. The experiments were repeated four times and error bars represent ± SD (**p < 0.005).

**Figure 5:**
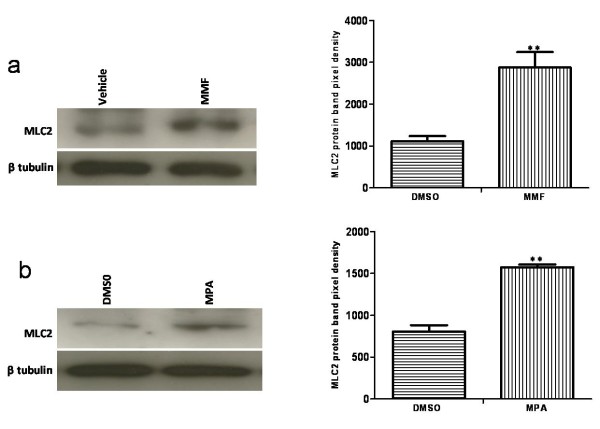
**Expression of MLC2 in MMF treated rat kidney lysate and HT-29 cells**. Protein lysate was prepared and immunoblotted for MLC2 as described in method section. β tubulin was used to show equal protein load. Lab image software was used for quantification of protein bands. Four independent experiments were performed and results presented as mean ± SD (**p < 0.005).

In the present study the alteration in the cellular proteome by the MPA treatment was investigated using HEK-293 as cell culture model. Incubation of HEK-293 cells with MPA followed a dose dependent inhibition of cell proliferation (Figure 1). The IC_50 _concentration (7.5 μmol/L or 2.4 mg/L) of MPA was selected as standard dose for further analysis, which is within the therapeutic range (0.3 to 3.4 mg/L) [14]. Cells were treated with MPA and DMSO (as vehicle) for 3 days, and total cell lysates were prepared. Total protein extracts of MPA and DMSO treated cells were separated by 2-DE using pH 3-10 linear IPG strips and visualized by silver stain. The protein spots which showed ≥ ± 1.5 fold change (p < 0.05 using Student's t test) as compared to DMSO treated controls were considered as differentially expressed proteins. Statistical analysis showed that a total of 12 proteins exhibited significantly altered expression due to MPA treatment (Table 1). The altered expression pattern of the HEK-293 proteins by MPA is shown in Figure 2. Among 12 regulated proteins spot under MPA treatment, 7 proteins were significantly up-regulated and 5 proteins showed down-regulated expression. The up-regulated spots under MPA treatment were identified as complement component 1Q subcomponent binding protein (C1q), electron transfer flavoprotein subunit beta, cytochrome b-c1 complex subunit, thioredoxin domain-containing protein 12, myosin regulatory light chain 2 (MLC2), peroxiredoxin1 (Prdx1) and profilin 1. Five proteins, which showed down-regulated expression, were identified as protein SET, stathmin, 40S ribosomal protein S12, histone H2B type 1-A, and histone H2B type 1-C/E/F/G/I. A bar diagram, showing relative abundance (% Vol), SD and statistical significance of all the significantly regulated protein is provided as "Additional file 1 figure s1". Figure 2 shows an exemplary gel of DMSO (vehicle) and MPA with marked regulated proteins. The extent of regulation in protein expression with predicted and actual pI, as well as molecular masses with their SwissProt accession numbers are provided in Table 1 and MS/MS spectral information is provided in the "Additional file 2 table s1". Functional classification of differentially regulated proteins was done using KOGnitor, an online biological function annotation tool [15]. The proteins altered by MPA treatment belong to various categories i.e., cytoskeleton (26%), chromatin structure/dynamics and energy production/conversion (17% each) (Figure 3). Gels spot diagram of two selected protein spots (MLC2 and Prdx1) in 4 biological replicates are shown in Figure 4a. To validate the 2-DE results, the expression of MLC2 and Prdx1 were confirmed by Western blotting and real time PCR analysis. Expression of Prdx1 and MLC2 were up-regulated at both transcriptional (Figure 4b) and protein level (Figure 4c). Specifically, MPA increased MLC2 protein (Mean fold: +1.78, p < 0.005, n = 4, Western blotting) and mRNA expression (Mean fold: +2.25, p < 0.05, n = 4, real time PCR). Prdx1 expression was also up-regulated, both at protein level (Mean fold: +2.73, p < 0.005, n = 4) and mRNA level (Mean fold: +1.93, p < 0.05, n = 4). To check whether over-expression of MLC2 following MPA treatment is only HEK-293 cells specific, we determined MLC2 expression in total protein lysate prepared from kidney of MMF (pro-drug of MPA) treated rats (Figure 5a) and MPA treated HT-29 cells (Figure 5b). MLC2 expression was increased both in kidney total protein lysate and HT-29 cells by (Mean fold: +2.57, p < 0.005, n = 4) and (Mean fold: +1.95, p < 0.005, n = 4) respectively.

**Table 1:** Differentially regulated proteins by MPA in HEK-293 cells identified by mass spectrometry

Spot No	Acc	M_t_/M_o_(kDa)	Score	pI_t_/pI_o_	Pep	Protein name	FunctionBy KOGnitor NCBI	Expression change (in folds)
6	Q01105	33.4/37.0	154	4.23/4.14	3	Protein SET	Replication, recombination and repair	1.86*↓
9	Q07021	31.3/31.0	141	4.74/4.5	3	Complement component 1 Q subcomponent-binding protein, mitochondrial	Defense mechanisms	1.58*↑
14	P38117	27.8/25.5	181	8.24/9.18	6	Electron transfer flavoprotein subunit beta	Energy production and conversion	1.54*↑
15	P47985	29.6/25.0	112	8.51/7.081	6	Cytochrome b-c1 complex subunit Rieske, mitochondrial	Energy production and conversion	3.71**↑
18	Q06830	22.0/21.0	64	8. 27/8.14	2	Peroxiredoxin-1	Posttranslational modification, protein turnover, chaperones	1.71**↑
22	P16949	17.2/15.8	56	5.76/6.32	3	Stathmin	General function prediction only	1.50**↓
23	O95881	19.1/16.0	123	5.24/5.89	4	Thioredoxin domain-containing protein 12	Cytoskeleton	1.95*↑
24	O14950	19.7/16.0	195	4.71/5.32	4	Myosin regulatory light chain MRLC2	Cytoskeleton	3.41*↑
27	Q96A08	14.1/14.5	51	10.31/7.0	2	Histone H2B type 1-A	Chromatin structure and dynamics	1.90*↓
28	P62807	13.8/14.2	250	10.31/6.51	9	Histone H2B type 1-C/E/F/G/I	Chromatin structure and dynamics	1.58*↓
31	P25398	14.5/13.0	89	6.81/7.10	3	40 S ribosomal protein S12	Translation, ribosomal structure and biogenesis	2.44*↓
34	P07737	15.0/13.5	142	8.44/9.07	6	Profilin-1	Cytoskeleton	1.51**↑

Acc: Accession number; Mt: theoretical molecular mass; Mo: observed molecular mass; pIt: theoretical isoelectric point; pIo: observed isoelectric point; pep: number of peptides sequenced for identification; Score: Peptide mass fingerprint probability score as defined by Mascot (www.matrixscience.com). Individual ions score > 42 indicate identity or extensive homology (p < 0.05); ↓: down-regulated; ↑ up-regulated; *p < 0.05, **p < 0.005. Molecular function determined from the online protein reference database KOGnitor NCBI (http://www.ncbi.nlm.nih.gov/COG/grace/kognitor.html).

**Figure 6:**
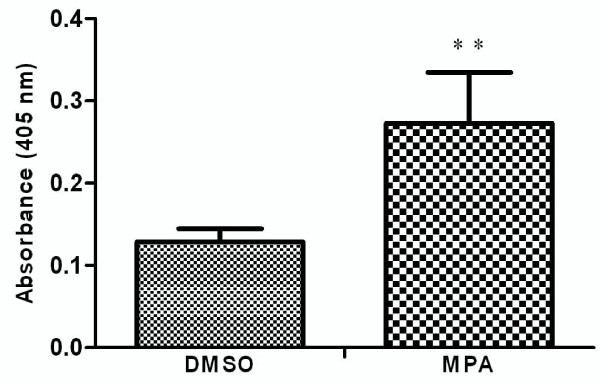
**Measurement of MPA induced caspase-3 activity**. Cells were treated with MPA and DMSO for 72 h. Protein extracts from each was measured for caspase-3 activity. Five independent experiments were performed and results presented as mean absorbance ± SD (**p < 0.005).

To demonstrate the effect of MPA on cell apoptosis, caspase-3 activity (apoptosis marker) was determined using a commercially available colorimetric assay. There was a significant difference in caspase-3 activity between MPA and DMSO treatment groups. MPA increased mean absorbance by 2 fold (p < 0.005, n = 5) as compared to DMSO treated cells. The results from caspase-3 assay revealed that MPA treated cells exhibit more apoptosis than cells treated with DMSO alone (Figure 6).

## Discussion

We have used a 2-DE and mass spectrometric based proteomics approach to develop a better understanding of the influence of MPA therapeutic dose on the proteome in HEK-293 cells. HEK-293 cells are widely used cell culture model to study the mechanisms of drug action, investigating drug targets and molecular aspects of xenobiotic toxicity [16-18]. The regulated proteins are found to be involved in diverse functions including apoptosis and cell signaling mechanism. Apoptosis assay showed that MPA has a pro-apoptotic role in HEK-293 cell line, a property which makes it a drug with potential anti-tumor activities. MLC2 is an important myosin regulatory subunit, which regulates smooth muscle and nonmuscle cells contractile activity [19]. MLC2 displayed an increased expression by MPA treatment. It is already reported that MPA influences the cellular cytoskeletal architecture via modulating mesangial actin reorganization by activating actin polymerization and inhibiting actin-depolymerization [20,21]. Phosphorylation of MLC2 causes significant changes in the physiological dynamics of actin cytoskeleton, leading to barrier defects in intestine [22], heart [23] and lungs [24]. However, it remains unclear if such cytoskeleton reorganization in different organs may lead to a completely different outcome, for example in intestine, diarrhoea is associated with MPA therapy in some patients [12]. In the present study, we observed that MLC2 over-expression is not limited to a specific cell type (i.e. HEK-293) but was reproducible in MMF treated rat kidney and in MPA treated HT-29 cells protein lysates.

We observed an increase Prdx1 expression by MPA treatment, both at gene and protein level.. Prdx1 is a cytoplasmic stress-inducible anti-oxidant enzyme and a major member of peroxiredoxin family [25]. Cells deficient in Prdx1 have increased sensitivity to oxidative DNA damage [26]. Prdx1 along with its anti-oxidant activity also possesses anti-inflammatory and anti-atherogenic effects [27]. Oxidative stress contributes to the pathophysiology of diverse clinical conditions, including ischemia-reperfusion mediated post transplantation graft injuries [28]. Prdx1 expression was also reported to be up-regulated in human gingival fibroblasts by cyclosporine A (another commonly used immunosuppressive drug) treatment [29]. MPA has previously been reported to diminish oxidative injuries and induce anti-oxidant effects by preventing the production of reactive oxygen species [10]. Furthermore, MPA exerts lesser oxidative stress in renal transplant patients, as compared to everolimus, cyclosporine and other calcineurin inhibitors [30,31] 30,31. Prdx1 contribute to the inhibition of tumorigenesis through PTEN/Akt pathway [32] and its lower expression in the tumor indicated high tumor proliferation, increased metastasis and could be used as cancer biomarker [33]. Prdx1 is also involved in ageing process as Prdx1-deficient mice have a shortened lifespan and other malignancies [26]. Anti-tumor drugs like histone deacetylase inhibitors (HDACIs) activate Prdx1, a tumor suppressor, which leads to apoptosis [34]. Previously it was observed that MPA also inhibit histone deacetylases (HDACs) [35]. A further investigation is needed to gain a deeper insight into the Prdx1 regulation by MPA through HDACs inhibition interaction with Prdx1 and its role in anti-tumor activities.

Profilin 1, another cytoskeletal protein was up-regulated by MPA treatment. Profilins are widely distributed actin binding proteins [36], involved in actin filament dynamics and several signaling pathways [37]. Profilin 1 over-expression has been reported to cause cell proliferation inhibition, apoptosis induction and tumor suppression [38]. Whether MPA via profilin over-expression exerts extended anti-proliferative or anti-tumor activities requires further investigation. Stathmin was down regulated by MPA. Stathmin is a 19 kDa cytoplasmic protein, which plays an important role in the regulation of the microtubule cytoskeleton. Stathmin regulates microtubule turnover by promoting microtubules depolymerization and hydrolyze guanosine triphosphate (GTP) from terminal tubulin, preventing polymerization of tubulin heterodimers [39]. Previously, our group demonstrated that AcMPAG alters tubulin polymerization in a concentration-dependent manner [40]. Furthermore, stathmin repression stabilizes microtubules, inhibits angiogenesis [41] and suppress tumors [42].

Thioredoxin domain-containing protein 12, also known as endoplasmic reticulum resident protein 18 (ERp18) is ubiquitous in mammalian cells and acts as a disulfide isomerase in the endoplasmic reticulum (ER). It provides defense against oxidative stress, refolds disulfide-containing proteins, and regulates transcription factors [43]. ERp18 expressional up-regulation might cause cell adoptivity in response to MPA induced ER stress. SET protein was down-expressed by MPA. SET, a major cellular serine threonine phosphatase is a potent inhibitor of protein phosphatase 2A (PP2A) activity [44] and a negative regulator of histone acetylation [45], thus involved in cell growth and signaling cascades [46]. PP2A expression induced by down-regulation of SET leads to the apoptosis and growth suppression [47],

MPA triggers nuclear stress and causes disruption of the nucleus, leading to the activation of p53, which may initiate cell cycle arrest and apoptosis [48]. In the present study histone H2B was down-regulated by MPA treatment, which is a major component of eukaryotic nucleosome core. Post translational modification such as methylation, acetylation, phosphorylation and ubiquitination of histone proteins alter transcription, DNA replication, and DNA repair [49,50]. Previous data showed that MPA mediated down-regulation of HDAC2 which might relate with potential epigenetic regulations [35]. The microrarray analysis of mononuclear cells treated with AcMPAG (a metabolite of MPA) showed down-regulation of histones in a previous study by our group [51].

MPA affects ribosomal machinery by decreasing intracellular guanine nucleotide level, depending on dosage and cell type, resulting in global reduction of RNA synthesis [48]. Other studies suggested that guanine nucleotide depletion by IMPDH leads to a decrease in pre-ribosomal RNA synthesis, nuclear disruption, and p53 activation [52]. Disorganization of nuclear and ribosomal biogenesis is suggested to be an effective therapeutic target in cancers [53]. We observed a down-regulation of 40 S ribosomal protein S12 by MPA, which might be due to the altered ribosome biogenesis. The proapoptotic stimuli including chemotherapeutic agents induced a dose-dependent increase in the expression of the cytochrome c proteins [54]. In the present study we also observed up-regulation of cytochrome b-c I complex by MPA which suggests a possible role of MPA in the regulation of energy metabolism. Complement component 1 Q subcomponent-binding protein (C1q), a component of complement system involved in the clearance of apoptotic cells was up-regulated by MPA. C1q binds to surface blebs of apoptotic cells, which follows subsequent phagocytosis [55]. C1q deficiency leads to a significant decline in the clearance of apoptotic cells in both C1q- and C4-deficient mice, causing glomerulonephritis [56]. MPA causes cellular apoptosis and cells might utilize C1q over-expression to clear the apoptotic cells.

## Conclusion

This investigation identifies proteins related to diverse cellular functions which altered their expression by MPA treatment; many of which are reported for the first time in this context. The expression of Prdx1 (involved in apoptosis) and MLC2 (protein important for epithelial barrier integrity) were observed to be regulated at RNA and protein level. Further investigations of the regulated proteins will provide new insights into the cellular pathways influenced by MPA therapy and could help in more rational use of MPA in transplantation medicine.

## Methods

### Reagents

Cell culture media (DMEM and MacCoy's), fetal calf serum (FCS), phosphate buffer saline (PBS), penicillin and streptomycin were purchased from PAA Laboratories, Colbe, Germany. Urea, thiourea, dithiothreitol (DTT), trypsin, triflouroacetic acid (TFA), sodium carbonate, ammonium bicarbonate, MPA and DMSO were purchased from Sigma-Aldrich, Steiheim, Germany. Acetonitril (ACN) was obtained from Promochem, Wasel, Germany. CHAPS was obtained from AppliChem, Darmstadt, Germany. Ampholytes, protein assay kit and immobilised pH gradient strips (IPG strips) were procured from Bio-Rad, Munich, Germany, while protease and phosphatase inhibitor cocktails were purchased from Roche, Mannheim, Germany. Bromophenol blue and trizma base were obtained from Carl Roth, Karlsruhe, Germany. Sodium dodecyl sulfate (SDS) was obtained from Serva, Heidelberg, Germany. Glycerin, potassium ferricynaide and sodium thiosulfate were purchased from Merck, Darmstadt, Germany and formic acid from BASF, Ludwigshafen, Germany.

### Cell culturing

HEK-293 and HT-29 cell lines were purchased from German collection of microorganisms and cell cultures (DSMZ), Braunschweig, Germany. The cells were grown in 75 cm^2 ^culture flasks (Sarstedt, Nuemberecht, Germany) and maintained in culture at 37°C in 95% humidity, 20% O_2 _and 5% CO_2. _DMEM and MacCoy's media supplemented with L-glutamine, 10% fetal calf serum, 100 U/mL penicillin, and 0.1 mg/mL streptomycin was used to grow HEK-293 and HT-29 cells respectively.

### Proliferation assay

Briefly, cells were grown in 96 well plates at a density of 3.5 × 10^4 ^cells/well at least 24 h prior to the start of the experiment. The cells were then incubated with DMSO (control) or 0 to 100 μmol/L MPA for a period of 72 h. After completion of incubation, proliferation was determined using ELISA based BrdU cell assay (Roche Diagnostics) according to manufacturer's recommendations. Four independent experiments were performed. IC_50 _values were calculated by a Grafit software package, version 5 (Erithacus Software, London, UK).

### Sample preparation for proteome analysis

The HEK-293 and HT-29 cells were grown for 24 h followed by treatment with DMSO or MPA (7.5 μmol/L and 10 μmol/L for HEK-293 and HT-29 respectively) for 72 h. Cells were harvested by scraping and were washed three times with ice cold PBS. After washing, cells were pelleted down at 250 × g for 10 min and lysed in a buffer containing 7 mol/L urea, 2 mol/L thiourea, 4% w/v CHAPS, 2% ampholyte pH 3-10 and 1% DTT. The lysates were centrifuged and protein content was measured by Bradford assay [57] using Bio-Rad protein reagent (Bio-Rad, Munich, Germany) according to manufacturer's instructions. Sample aliquots were kept at -80°C until further use. Protein lysate was prepared from 21 days MMF treated adult female Wistar rat's kidney according to the previously reported protocol [58] and were used for Westernblotting.

### 2-DE

The 2-DE was performed as described by Gorg et al 2000 [59] with some minor modifications. Protein samples of HEK-293 cell (110 μg) were mixed with rehydration buffer (7 mol/L urea, 2 mol/L thiourea, 4% CHAPS, 0.2% ampholyte [pH 3-10], and 0.2% DTT) containing trace amount of bromophenol blue to a total volume of 350 μL. Samples were applied to linear IPG strips [pH 3-10], Bio- Rad) for 1 h and then covered with mineral oil for passive rehydration overnight at room temperature. Iso-electric focusing (IEF) was performed in Protean IEF cell (Bio-Rad) with a program of 1 h at 100 volts, 1 h at 500 volts, 2 h at 1000 volts and 8000 volts with a total of 32000 volts-h. For the second dimension electrophoretic separation, focused strips were equilibrated for 30 min at room temperature in a buffer containing 50 mmol/L Tris-HCL [pH 8.8], 6 mol/L urea, 30% v/v glycerol, 2% SDS and 10 g/L DTT followed by an identical incubation but replacing DTT with 40 g/L iodoacetamide. The proteins in the equilibrated strips were then resolved on the 12.5% SDS-PAGE in a Protean II chamber (Bio-Rad) at 100 V/4°C.

### Protein visualization, densitometric analysis and in-gel digestion

Gels were silver stained as described by Blum et al 1987 [60]. After fixation, gels were washed and sensitized. The gels were then incubated in freshly prepared silver nitrate solution (0.2% silver nitrate and 0.026% formaldehyde) for 20 min at room temperature followed by 3 times washes of 20 sec each in distilled water. Gels were placed in developing solution (6% sodium carbonate, 0.018526% formaldehyde and 6% sodium thiosulfate) until standard marker stained completely and adequate spots were visualized. Gels were scanned with a gel Cano scan 8400 (Canon, Tokyo, Japan). Densitrometric analysis was done by using Delta 2D software version 3.6 (Decodon GmbH, Gerifswald, Germany) [61]. Spot intensities were first normalized and the relative intensity of each spot was calculated by dividing the intensity of each spot by the sum of all spots intensities on the corresponding gel. Fold change, SD and Student's t test probability were calculated using Microsoft excel software. Spots having at least 1.5 fold expressional changes (p < 0.05) were considered statistically significant. Four independent 2-DE experiments were performed. Differentially regulated protein spots were excised from the silver stained gel with a clean scalpel blade followed by in-gel digestion according to the method adopted and modified from Shevchenko et al [62]. Briefly, the gel pieces were washed twice in 100 mmol/L ammonium bicarbonate/acetonitrile (1:1, v/v) initially for 10 min and then until all visible dye was removed. The gel pieces were dried using vacuum centrifuge (UNIVAPO 150 H; uniEquip, Matinsried, Germany) followed by reconstitution in the trypsin digestion solution (10 ng/μL in 100 mmol/L ammonium bicarbonate) overnight at 37°C. After incubation the supernatant containing digested peptides was transferred to a tube and 50 μL of 0.1% TFA was added followed by sonication for 30 min. After sonication, the supernatant was pooled with the previous one. Two further extractions were collected in the same way using 0.1% TFA in 30% and then 60% ACN. The pooled extracts of peptides were dried in vacuum centrifuge and reconstituted in 0.1% formic acid.

### Q-TOF LC-MS/MS analysis of protein identification

The reconstituted peptide samples (1 μL) were introduced onto μ-precolumn™ cartridge (C18 pepMap; 300 μm × 5 mm; 5 μm particle size) and further separated through a C18 pepMap 100 nano- Series™ (75 μm × 15 cm; 3 μm particle size) analytical column (LC Packings, Germering, Germany) using an CapLC autosampler (Waters, Eschborn, Germany). The mobile phase consisted of solution A (0.1% formic acid prepared in 5% ACN) and solution B (0.1% formic acid prepared in 95% ACN). The sample run time was set to 60 min and the flow rate of the pump to 5 μL/min. The exponential gradient was initiated at 5 min after loading from 10% to 95% for the period of 50 min. Tip flow rate of 250 nL/min was achieved through a flow splitter. The eluted peptides were injected into a Q-TOF Ultima Global (Micromass, Manchester, UK) mass spectrometer equipped with a nanoflow ESI Z-spray source in positive ion mode. Data was acquired by MassLynx (v 4.0) software and peak list (pkl file) was generated from acquired MS/MS raw data using ProteinLynx Global Server bioinformatics tool (PLGS; v 2.2; Waters, Manchester, U.K.) under the following settings; Electrospray, centroid 80% with minimum peak width 4 channel, noise reduction 10%, Savitzky-Golay, MSMS, medium deisotoping with 3% threshold, no noise reduction and no smoothing. The generated pkl files were searched using the online MASCOT (http://www.matrixscience.com) algorithm against the SwissProt data base release 15.5 (515203 sequence entries, 181334896 elements). The search criteria was set as follows: enzyme, trypsin; allowance of up to one missed cleavage peptide; mass tolerance ± 0.5 Da and MS/MS tolerance ± 0.5 Da; modifications of cysteine carboamidomethylation and methionine oxidation. Proteins were finally identified on the basis of two or more peptides, whose ion scores exceeded the threshold, *P *< 0.05, which indicated the 95% confidence level for these matched peptides. To ensure accurate identification, protein spots were digested from more than two gels and analyzed with MS. Proteins were considered as identified if the threshold was exceeded and the protein spot possessed the correct molecular weight and pI value of the corresponding spot on 2-DE.

### Functional classification

Biological function annotations for all of the identified proteins were done by KOGnitor (http://www.ncbi.nlm.nih.gov/COG/grace/kognitor.html) [15].

### Western blotting

Proteins were separated on 12.5% SDS-PAGE and blotted onto PVDF membrane (ImmobilonP, Millipore) using semidry Trans-Blot^® ^SD cell system (Bio-Rad, Munich, Germany) for 30 min at 15 V in a blotting buffer (192 mmol/L glycine, 20% methanol, 25 mmol/L Tris, pH 8.3). The membranes were blocked with 5% (w/v) skimmed milk repared in TBS-T buffer (50 mmol/L Tris-HCl [pH 7.5], 200 mmol/L NaCl, 0.05% Tween 20) for 1 h at room temperature and washed twice with TBS-T buffer. The membranes were incubated with 1:1000 mouse anti Prdx1 antibody (Abcam, Cambridge, MA), 1:1000 rabbit anti MLC2 (Cell Signaling Technology, Inc., Danvers, MA) and 1:1000 mouse anti beta tubulin (Biovender, Czech Republic) overnight at 4°C, followed by washes with TBS-T buffer. Membranes were further incubated with appropriate HRP-conjugated secondary antibodies for 1 h at room temperature. The signals on the blots were detected by using ECL system (GE Healthcare) according to manufacturer's instructions. Signal intensities from each Western blot were quantified by using Lab Image software, version 2.71 (Leipzig, Germany). β tubulin was used as a loading control and at least four independent experiments were performed.

### RNA isolation and cDNA synthesis

RNA was extracted using Trizol reagent (Invitrogen, Carlsbad, CA) according to manufacturer's recommendations. Briefly, cells were scraped, washed and then homogenized in Trizol reagent. RNA was separated by chloroform/isopropanol precipitation method. The concentration of RNA was determined by the GeneQuant II RNA/DNA calculator (Pharmacia Biotech, Freiburg, Germany). The RNA quality was verified at OD_260_/OD_280 _nm ratios and subsequent electrophoretically on 1% agarose gels using ethidium bromide staining. The cDNAs were synthesized from 2 μg total RNA in a 30 μL reaction mix containing 1 × reverse transcriptase (RT) PCR buffer (10 mmol/L Tris-HCL [pH 8.3], 15 mmol/L KCl, 0.6 mmol/L MgCl_2_), 0.5 mmol/L of dNTPs mix, 1 U/μL RNase inhibitor and 13.3 U/μL M-MLV RT enzyme. The RT reaction was performed in a thermocycler (Biometra, Goettingen, Germany) at 42°C for 1 h. cDNA was stored at -70°C until use.

### Real-time PCR

Relative quantitative PCR were carried out using the LightCycler instrument (Roche Diagnostic Systems, NJ, USA). The primers for the human Prdx1 (forward 5'-TGGGGTCTTAAAGGCTGATG-3' and reverse 5'-TCCCCATGTTTGTCAGTGAA -3'), human MLC2 (forward 5'- CAGGAGTTCAAAGAGGCCTTCAAC -3' and reverse 5'- CTGTACAGCTCATCCACTTCCTCA -3') and elongation factor 2 (forward 5'-GACATCACCAAGGGTGTGCAG-3' and reverse 5'-GCGGTCAGCACACTGGCATA-3) were designed by the Primer3 software (http://frodo.wi.mit.edu) [63]. The total volume of 20 μL PCR contained 1 μL of cDNA solution, 2 μL of 10 × PCR buffer (Invitrogen), 2 μL Syber green, 1 μL BSA, 1 μL DMSO, 0.25 μL of each primer (Eurofins MWG-Biotech, Ebersberg, Germany), 2.0 mmol/L MgCl_2_, 0.2 mmol/L dNTPs mix and 0.15 U/μL PAN Script DNA polymerase (PAN Biotech, Aidenbach, Germany). The amplification conditions for Prdx1 and MLC2 were: initial denaturation 30 sec at 95°C and repeated cycles of denaturation (95°C for 1 sec), primer annealing (55°C for 5 sec), elongation (72°C for 10 sec), and fluorescence reading at 82°C. For elongation factor 2 (EF-2) PCR conditions were similar to Prdx1 except for primer fluorescence reading which was measured at 88°C. The relative expression of Prdx1 and MLC2 mRNA in the treated samples was determined as a fold increase compared with control samples using the comparative threshold cycle (C_T_) method 2^-ΔΔ C^_T_(ΔΔ*C*_T _= Δ*C *target genes - Δ*C *reference gene) [64]. EF-2 was used as the internal control gene. Experiments were performed four times. Statistical difference (p value) in mRNA expression level between MPA and DMSO samples were calculated using the Mann-Whitney U test. The PCR product was run on a 1% ethidium bromide-agarose gel to confirm the presence of desired specific amplified product.

### Apoptosis assay

The caspase 3 activity was measured using CaspACE™ Assay kit (Promega Corporation, WI, USA) according to the manufacturer's protocol. Cells were treated with DMSO and MPA for 72 h, harvested and briefly suspended in lysis buffer. Proteins were extracted and quantified by Bradford method [57]. Briefly, 70 μg of protein lysate were mixed with reaction mixtures containing colorimetric substrate peptides specific for caspase 3 (DEVD-pNA) and then incubated at room temperature for overnight. The absorbance of the cleaved *p*-nitroanilide from the substrate DEVD-*p*NA was measured at 405 nm using EL808 microplate reader (Bio-Tek instruments, VT, USA). Five independent experiments were performed.

## Competing interests

The authors declare that they have no competing interests.

## Authors' contributions

MQ carried out the experiments and drafted the manuscript. HR contributed to the data analysis. ARA and MO participated in the design, supervision and interpretation of the study. All authors have read and approved the final manuscript.

## Supplementary Material

Additional file 1 **A graphical representation of relative abundance (% volume) of all differentially regulated proteins**. Relative abundance of the proteins differentially expressed in DMSO and MPA treated HEK-293 cells. Results shown as mean of four independent experiments (*p < 0.05 or **p < 0.005). Click here for file

Additional file 2 **MS/MS analysis table of all differentially regulated proteins**. Accession number, sequence coverage, score and MS/MS spectra of identified proteins. Click here for file
