# Supplementary figures and images for: Differential proteome analysis of human embryonic kidney cell line (HEK-293) following mycophenolic acid treatment
Source: Proteome Sci. 2011 Sep 20;9:57. doi: 10.1186/1477-5956-9-57 (PMC3189873; doi:10.1186/1477-5956-9-57)

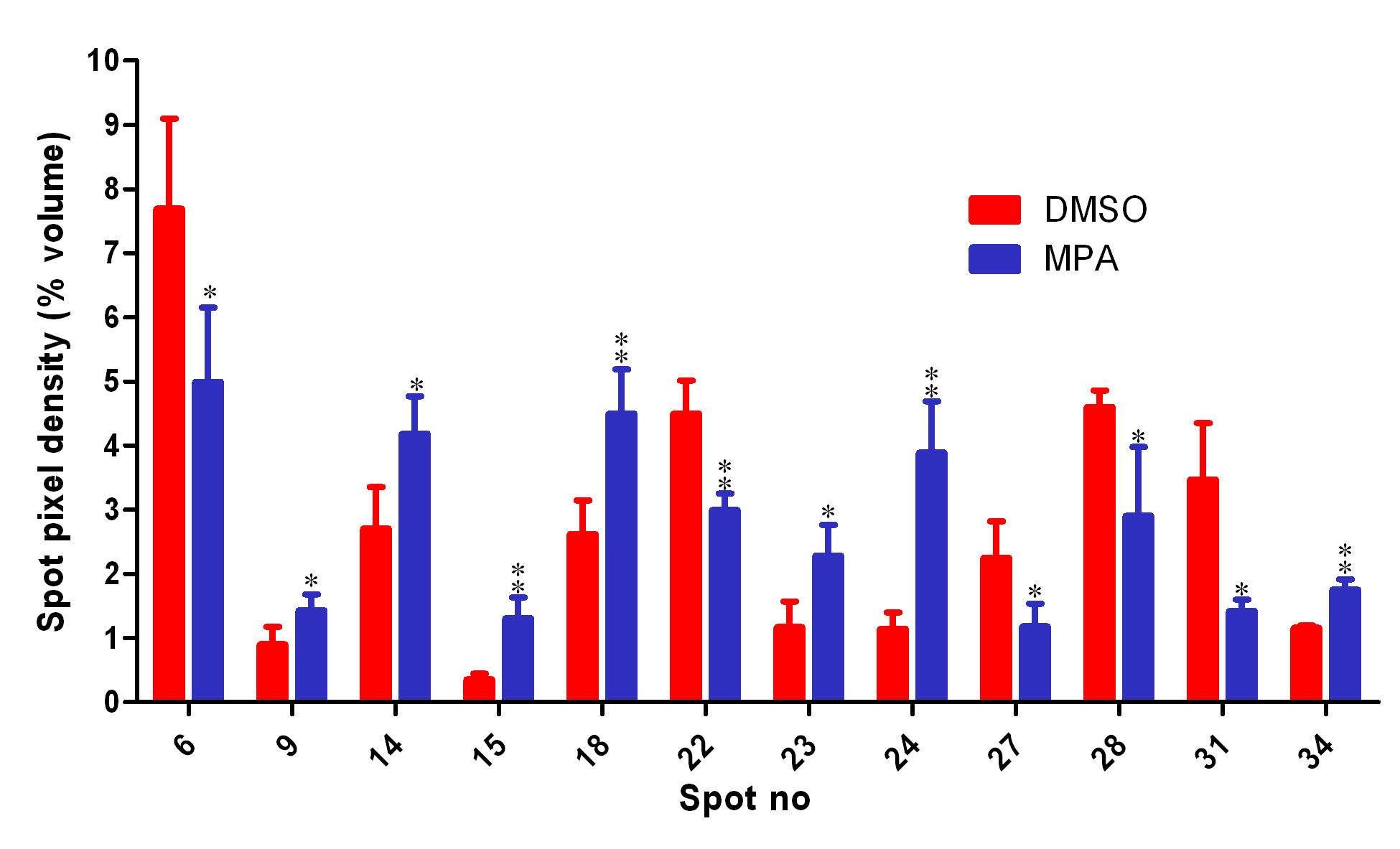

Supplement: Additional file 1 — A graphical representation of relative abundance (% volume) of all differentially regulated proteins. Relative abundance of the proteins differentially expressed in DMSO and MPA treated HEK-293 cells. Results shown as mean of four independent experiments (*p < 0.05 or **p < 0.005). [file 1477-5956-9-57-S1.TIFF]
