# Supplementary material for: Differential proteome analysis of human embryonic kidney cell line (HEK-293) following mycophenolic acid treatment
Source: Proteome Sci. 2011 Sep 20;9:57. doi: 10.1186/1477-5956-9-57 (PMC3189873; doi:10.1186/1477-5956-9-57)
Supplement: Additional file 2 — MS/MS analysis table of all differentially regulated proteins. Accession number, sequence coverage, score and MS/MS spectra of identified proteins. [file 1477-5956-9-57-S2.DOC]

**Supplementary Table:**

MS/MS analysis table for identified proteins

**Footnotes:** ^1^Mass 1 = Molecular mass of the protein observed in Mascot search. ^2^Mascot score 2 = >40 indicate identification or extensive homology

(p < 0.05). ^3^Peptide matched 3= Number of peptides matched with protein in MS/MS query. ^4^pI 4 = Isoeletric point of the protein observed in Mascot search.

| **Spot no.** | **Accession no.** | **Mass^1^ (kDa)** | **pI^4^** | **Mascot**  **Score^2^** | **Peptide matched^3^** | **Sequence Coverage** | **Protein name** | **MS/MS Analysis** |
| --- | --- | --- | --- | --- | --- | --- | --- | --- |
| 6 | Q01105 | 33.4 | 4.23 | 154 | 3 | 11 | Protein SET | **1** MAPKRQSPLP PQKKKPRPPP ALGPEETSAS AGLPKKGEKE QQEAIEHIDE  **51** VQNEIDR**LNE QASEEILK**VE QKYNKLRQPF FQKRSELIAK IPNFWVTTFV  **101** NHPQVSALLG EEDEEALHYL TR**VEVTEFED IK**SGYRIDFY FDENPYFENK  **151** VLSK**EFHLNE SGDPSSK**STE IKWKSGKDLT KRSSQTQNKA SRKRQHEEPE  **201** SFFTWFTDHS DAGADELGEV IKDDIWPNPL QYYLVPDMDD EEGEGEEDDD  **251** DDEEEEGLED IDEEGDEDEG EEDEDDDEGE EGEEDEGEDD  **Start-End Observed Mr(expt) Mr(calc) Delta Miss Sequence**  **58 - 68 637.3448 1272.6750 1272.6561 0.0190 0 R.LNEQASEEILK.V**  **123 - 132 604.8156 1207.6166 1207.5972 0.0194 0 R.VEVTEFEDIK.S**  **155 - 167 482.8923 1445.6551 1445.6423 0.0128 0 K.EFHLNESGDPSSK.S**  MS/MS Fragmentation of **VEVTEFEDIK**  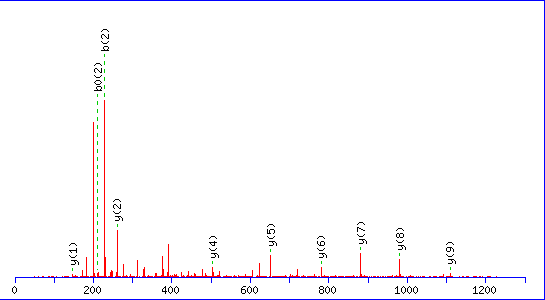 |
| 9 | Q07021 | 31.3 | 4.74 | 141 | 3 | 9 | Complement component 1 Q subcomponent-binding protein, mitochondrial | **1** MLPLLRCVPR VLGSSVAGLR AAAPASPFRQ LLQPAPRLCT RPFGLLSVRA  **51** GSERRPGLLR PRGPCACGCG CGSLHTDGDK AFVDFLSDEI KEERKIQKHK  **101** TLPK**MSGGWE LELNGTEAK**L VRKVAGEKIT VTFNINNSIP PTFDGEEEPS  **151** QGQKVEEQEP ELTSTPNFVV EVIKNDDGKK ALVLDCHYPE DEVGQEDEAE  **201** SDIFSIR**EVS FQSTGESEWK** DTNYTLNTDS LDWALYDHLM DFLADRGVDN  **251** TFADELVELS TALEHQEYIT FLEDLKSFVK SQ  **Start -End Observed Mr(expt) Mr(calc) Delta Miss Sequence**  **105-119 819.3414 1636.6682 1636.7403 -0.0720 0 K.MSGGWELELNGTEAK.L**   **208 - 220 757.3118 1512.6090 1512.6732 -0.0642 0 R.EVSFQSTGESEWK.**  **208 - 220 757.3123 1512.6100 1512.6732 -0.0632 0 R.EVSFQSTGESEWK.D**  MS/MS Fragmentation of **EVSFQSTGESEWK**  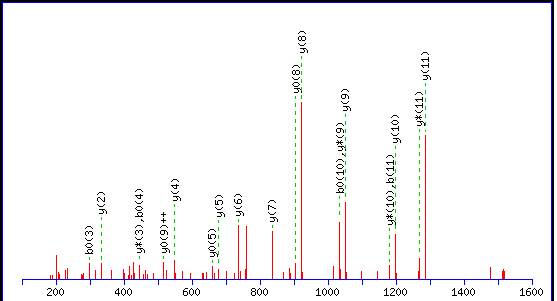 |
| 14 | P38117 | 27.8 | 8.24 | 181 | 6 | 22 | Electron transfer flavoprotein subunit beta | **1** MAELRVLVAV KRVIDYAVKI RVKPDR**TGVV TDGVK**HSMNP FCEIAVEEAV  **51** RLKEKKLVKE VIAVSCGPAQ CQETIR**TALA MGADR**GIHVE VPPAEAER**LG**  **101 PLQVAR**VLAK LAEKEKVDLV LLGKQAIDDD CNQTGQMTAG FLDWPQGTFA  **151** SQVTLEGDKL KVER**EIDGGL ETLR**LKLPAV VTADLRLNEP RYATLPNIMK  **201** AKKKKIEVIK PGDLGVDLTS K**LSVISVEDP PQR**TAGVK**VE TTEDLVAK**LK  **251** EIGRI  **Start-End Observed Mr(expt) Mr(calc) Delta Miss Sequence**  **27 - 35 438.2605 874.5064 874.4760 0.0304 0 R.TGVVTDGVK.H**  **77 - 85 461.2477 920.4808 920.4386 0.0423 0 R.TALAMGADR.G**  **99 - 106 427.2842 852.5538 852.5181 0.0357 0 R.LGPLQVAR.V**  **165 - 174 551.8152 1101.6158 1101.5666 0.0493 0 R.EIDGGLETLR.L**  **222 - 233 670.3985 1338.7824 1338.7143 0.0681 0 K.LSVISVEDPPQR.T**  **239 - 248 552.8170 1103.6194 1103.5710 0.0484 0 K.VETTEDLVAK.L**  MS/MS Fragmentation of **LSVISVEDPPQR**  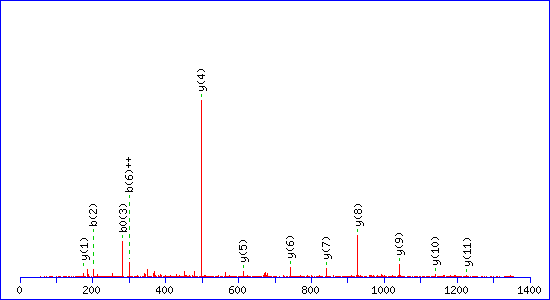 |
| 15 | P47985 | 29.6 | 8.51 | 112 | 6 | 16 | Cytochrome b-c1 complex subunit Rieske, mitochondrial | **1** MLSVASRSGP FAPVLSATSR GVAGALRPLV QATVPATPEQ PVLDLKRPFL  **51** SRESLSGQAV RRPLVASVGL NVPASVCYSH TDIK**VPDFSE YRRLEVLDST**  **101 K**SSRESSEAR KGFSYLVTGV TTVGVAYAAK NAVTQFVSSM SASADVLALA  **151** KIEIKLSDIP EGKNMAFKWR **GKPLFVR**HRT QK**EIEQEAAV ELSQLRDPQH**  **201 DLDR**VKKPEW VILIGVCTHL GCVPIANAGD FGGYYCPCHG SHYDASGRIR  **251** LGPAPLNLEV PTYEFTSDDM VIVG  **Start-End Observed Mr(expt) Mr(calc) Delta Miss Sequence**  **85 - 92 506.7550 1011.4954 1011.4662 0.0293 0 K.VPDFSEYR.R**  **93 - 101 530.8184 1059.6222 1059.5924 0.0298 1 R.RLEVLDSTK.S**  **94 - 101 452.7524 903.4902 903.4913 -0.0011 0 R.LEVLDSTK.S**  **171 - 177 408.7669 815.5192 815.5018 0.0175 0 R.GKPLFVR.H**  **183 - 196 807.9452 1613.8758 1613.8260 0.0498 0 K.EIEQEAAVELSQLR.D**  **197 - 204 498.2446 994.4746 994.4468 0.0278 0 R.DPQHDLDR.V**  MS/MS Fragmentation of **DPQHDLDR**  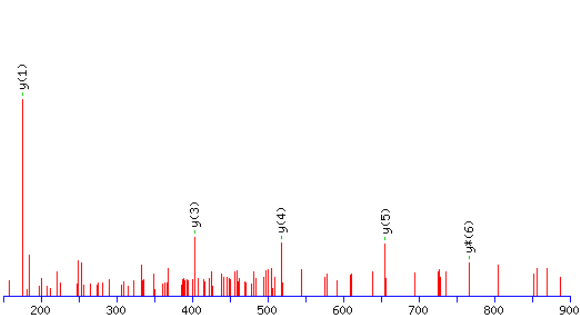 |
| 18 | Q06830 | 22.0 | 8.27 | 64 | 2 | 10 | Peroxiredoxin-1 | **1** MSSGNAKIGH PAPNFKATAV MPDGQFKDIS LSDYKGKYVV FFFYPLDFTF  **51** VCPTEIIAFS DRAEEFKKLN CQVIGASVDS HFCHLAWVNT PKKQGGLGPM  **101** NIPLVSDPKR **TIAQDYGVLK** ADEGISFRGL FIIDDKGILR **QITVNDLPVG**  **151 R**SVDETLRLV QAFQFTDKHG EVCPAGWKPG SDTIKPDVQK SKEYFSKQK  **Start-End Observed Mr(expt) Mr(calc) Delta Miss Sequence**  **111 - 120 554.2855 1106.5564 1106.5972 -0.0407 0 R.TIAQDYGVLK.A**  **141 - 151 606.3233 1210.6320 1210.6670 -0.0349 0 R.QITVNDLPVGR.S**  MS/MS Fragmentation of **TIAQDYGVLK.**  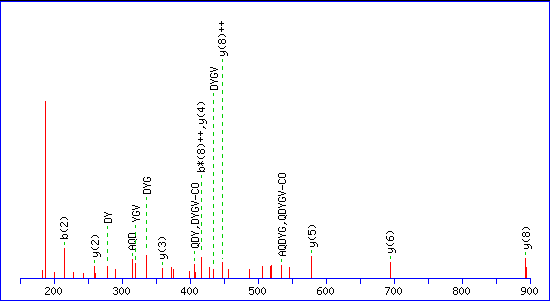 |
| 22 | P16949 | 17.2 | 5.76 | 56 | 3 | 18 | Stathmin | **1** MASSDIQVKE LEKRASGQAF ELILSPRSKE SVPEFPLSPP KKK**DLSLEEI**  **51 QKKLEAAEER** RKSHEAEVLK QLAEKREHEK EVLQK**AIEEN NNFSK**MAEEK  **101** LTHKMEANKE NREAQMAAKL ERLREKDKHI EEVRKNKESK DPADETEAD  **Start-End Observed Mr(expt) Mr(calc) Delta Miss Sequence**  **44 - 52 537.7797 1073.5448 1073.5604 -0.0156 0 K.DLSLEEIQK.**  **53 - 60 473.2399 944.4652 944.4927 -0.0274 1 K.KLEAAEER.R**  **86 - 95 583.2628 1164.5110 1164.5411 -0.0300 0 K.AIEENNNFSK.M**  MS/MS Fragmentation of **AIEENNNFSK**  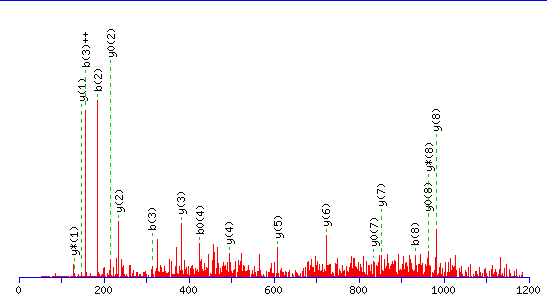 |
| 23 | O95881 | 19.1 | 5.24 | 123 | 4 | 13 | Thioredoxin domain-containing protein 12 | **1** METRPRLGAT CLLGFSFLLL VISSDGHNGL GKGFGDHIHW RTLEDGKKEA  **51** AASGLPLMVI IHKSWCGACK ALKPKFAEST EISELSHNFV MVNLEDEEEP  **101** KDEDFSPDGG YIPR**ILFLDP SGK**VHPEIIN ENGNPSYK**YF YVSAEQVVQG**  **151 MK**EAQERLTG DAFRKKHLED EL  **Start-End Observed Mr(expt) Mr(calc) Delta Miss Sequence**  **115 - 123 495.3159 988.6172 988.5593 0.0579 0 R.ILFLDPSGK.V**  **115 - 123 495.3221 988.6296 988.5593 0.0703 0 R.ILFLDPSGK.V**  **139 - 152 832.9714 1663.9282 1663.7916 0.1367 0 K.YFYVSAEQVVQGMK.E**   **139 - 152 832.9721 1663.9296 1663.7916 0.1381 0 K.YFYVSAEQVVQGMK.E**  MS/MS Fragmentation of **YFYVSAEQVVQGMK**  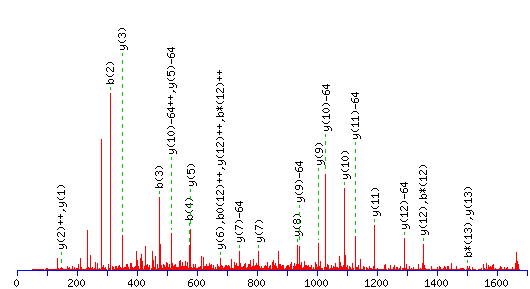 |
| 24 | O14950 | 19.7 | 4.71 | 195 | 4 | 23 | Myosin regulatory light chain MRLC2 | **1** MSSKKAKTKT TKKRPQRATS NVFAMFDQSQ IQEFKEAFNM IDQNRDGFID  **51** KEDLHDMLAS LGKNPTDAYL DAMMNEAPGP INFTMFLTMF GEK**LNGTDPE**  **101 DVIR**NAFACF DEEATGTIQE DYLR**ELLTTM GDRFTDEEVD ELYR**EAPIDK  **151** K**GNFNYIEFT R**ILKHGAKDK DD  **Start - End Observed Mr(expt) Mr(calc) Delta Miss Sequence**  **94 - 104 614.8414 1227.6682 1227.6095 0.0587 0 K.LNGTDPEDVIR.N**  **125 - 133 526.2847 1050.5548 1050.5016 0.0533 0 R.ELLTTMGDR.F**  **134 - 144 708.3533 1414.6920 1414.6252 0.0668 0 R.FTDEEVDELYR.E**  **152 - 161 630.8325 1259.6504 1259.5935 0.0570 0 K.GNFNYIEFTR.I**  MS/MS Fragmentation of **LNGTDPEDVIR**  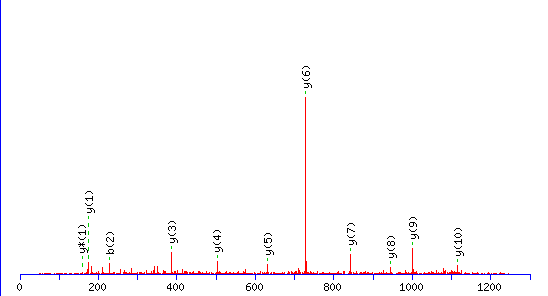 |
| 27 | Q96A08 | 14.1 | 10.31 | 51 | 2 | 12 | Histone H2B type 1-A | **1** MPEVSSKGAT ISKKGFKKAV VKTQKKEGKK RKRTRKESYS IYIYKVLKQV  **51** HPDTGISSKA MSIMNSFVTD IFERIASEAS RLAHYSKRST ISSR**EIQTAV**  **101 RLLLPGELAK** HAVSEGTKAV TKYTSSK  **Start - End Observed Mr(expt) Mr(calc) Delta Miss Sequence**  **95 - 101 408.7352 815.4558 815.4501 0.0057 0 R.EIQTAVR.L**  **102 - 110 477.3018 952.5890 952.5957 -0.0066 0 R.LLLPGELAK.**  MS/MS Fragmentation of **LLLPGELAK**  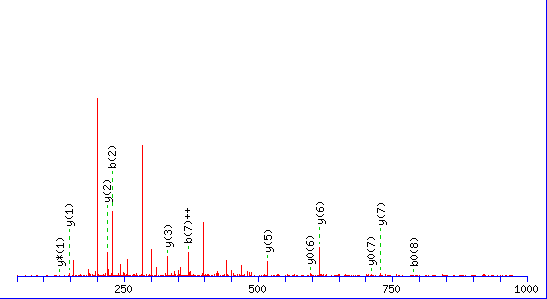 |
| 28 | P62807 | 13.8 | 10.31 | 250 | 9 | 57 | Histone H2B type 1-C/E/F/G/I | **1** MPEPAKSAPA PKKGSKKAVT KAQKKDGKKR KRSRK**ESYSV YVYKVLKQVH**  **51 PDTGISSKAM GIMNSFVNDI FER**IAGEASR LAHYNKR**STI TSREIQTAVR**  **101 LLLPGELAKH AVSEGTKAVT K**YTSK  **Start - End Observed Mr(expt) Mr(calc) Delta Miss Sequence**  **36 - 44 569.2944 1136.5742 1136.5390 0.0353 0 K.ESYSVYVYK.V**  **45 - 58 503.6217 1507.8433 1507.8358 0.0074 1 K.VLKQVHPDTGISSK.A**  **48 - 58 584.8140 1167.6134 1167.5884 0.0250 0 K.QVHPDTGISSK.A**  **59 - 73 888.4241 1774.8336 1774.8018 0.0318 0 K.AMGIMNSFVNDIFER.I**  **88 - 100 487.9460 1460.8162 1460.7947 0.0215 1 R.STITSREIQTAVR.L**  **94 - 100 408.7277 815.4408 815.4501 -0.0093 0 R.EIQTAVR.L**  **101 - 109 477.2920 952.5694 952.5957 -0.0262 0 R.LLLPGELAK.H**  **101 - 109 477.3095 952.6044 952.5957 0.0088 0 R.LLLPGELAK.H**  **110 - 121 409.8952 1226.6638 1226.6619 0.0019 1 K.HAVSEGTKAVTK.Y**  MS/MS Fragmentation of **STITSREIQTAVR**  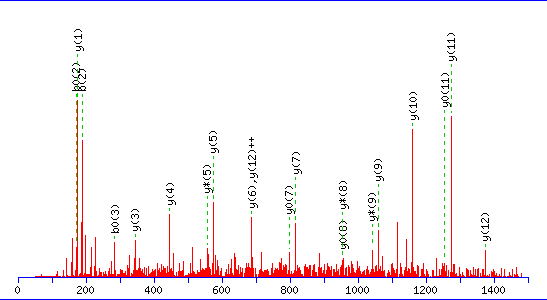 |
| 31 | P25398 | 14.5 | 6.81 | 89 | 3 | 14 | 40S ribosomal protein S12 | **1** MAEEGIAAGG VMDVNTALQE VLK**TALIHDG LAR**GIREAAK ALDKRQAHLC  **51** VLASNCDEPM YVKLVEALCA EHQINLIKVD DNKK**LGEWVG LCK**IDREGKP  **101** RKVVGCSCVV VKDYGKESQA KDVIEEYFKC KK  **Start - End Observed Mr(expt) Mr(calc) Delta Miss Sequence**  **24 - 33 533.7745 1065.5344 1065.5931 -0.0586 0 K.TALIHDGLAR.G**  )  **24 - 33 533.7747 1065.5348 1065.5931 -0.0582 0 K.TALIHDGLAR.G**  **85 - 93 531.2432 1060.4718 1060.5376 0.0657 0 K.LGEWVGLCK.I**  MS/MS Fragmentation of **LGEWVGLCK**  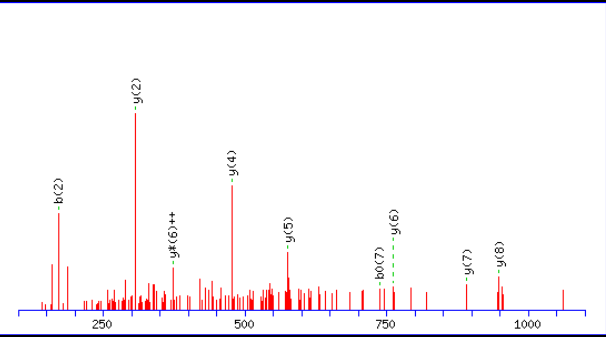 |
| 34 | P07737 | 15.0 | 8.44 | 142 | 06 | 53 | Profilin-1 | **1** MAGWNAYIDN LMADGTCQDA AIVGYKDSPS VWAAVPGK**TF VNITPAEVGV**  **51 LVGK**DRSSFY VNGLTLGGQK CSVIR**DSLLQ DGEFSMDLR**T K**STGGAPTFN**  **101 VTVTKTDKTL VLLMGKEGVH GGLINKKCYE MASHLR**RSQY  **Start - End Observed Mr(expt) Mr(calc) Delta Miss Sequence**  **39 - 54 822.4751 1642.9356 1642.9294 0.0062 0 K.TFVNITPAEVGVLVGK.D**  **76 - 89 813.3810 1624.7474 1624.7403 0.0072 0 R.DSLLQDGEFSMDLR.T**  **92 - 105 690.3588 1378.7030 1378.7093 -0.0062 0 K.STGGAPTFNVTVTK.**  **106 - 116 609.8624 1217.7102 1217.7053 0.0049 1 K.TDKTLVLLMGK.E**  **117 - 127 576.3310 1150.6474 1150.6458 0.0016 1 K.EGVHGGLINKK.C**  **128 - 136 583.7573 1165.5000 1165.5008 -0.0008 0 K.CYEMASHLR.R**  MS/MS Fragmentation of **TFVNITPAEVGVLVGK**  **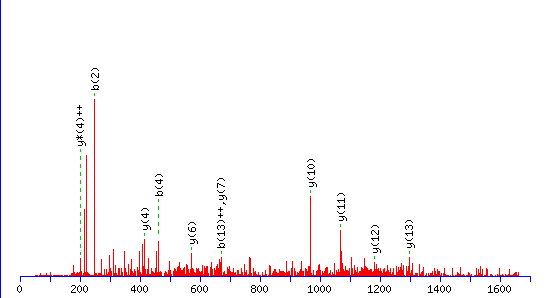** |
